# Endophytic fungi: A future prospect for breast cancer therapeutics and drug development

**DOI:** 10.1016/j.heliyon.2024.e33995

**Published:** 2024-07-04

**Authors:** Sherin Varghese, M.S. Jisha, K.C. Rajeshkumar, Virendra Gajbhiye, Abdulwahed Fahad Alrefaei, Rajesh Jeewon

**Affiliations:** a School of Biosciences, Mahatma Gandhi University, Kottayam, Kerala, 686560, India; b National Fungal Culture Collection of India (NFCCI), Biodiversity and Palaeobiology (Fungi) Gr., Agharkar Research Institute, G.G. Agharkar Road, Pune, 411 004, Maharashtra, India; c Nanobioscience Group, Agharkar Research Institute, G.G. Agharkar Road, Pune, 411 004, Maharashtra, India; d Department of Zoology, College of Science, King Saud University, P.O. Box 2455, Riyadh, 11451, Saudi Arabia; e Department of Health Sciences, Faculty of Medicine and Health Sciences, University of Mauritius, Reduit, Mauritius

**Keywords:** Anticancer compounds, Breast cancer– endophytic fungi –medicinal plants

## Abstract

Globally, breast cancer is a primary contributor to cancer-related fatalities and illnesses among women. Consequently, there is a pressing need for safe and effective treatments for breast cancer. Bioactive compounds from endophytic fungi that live in symbiosis with medicinal plants have garnered significant interest in pharmaceutical research due to their extensive chemical composition and prospective medicinal attributes. This review underscores the potentiality of fungal endophytes as a promising resource for the development of innovative anticancer agents specifically tailored for breast cancer therapy. The diversity of endophytic fungi residing in medicinal plants, success stories of key endophytic bioactive metabolites tested against breast cancer and the current progress with regards to *in vivo* studies and clinical trials on endophytic fungal metabolites in breast cancer research forms the underlying theme of this article. A thorough compilation of putative anticancer compounds sourced from endophytic fungi that have demonstrated therapeutic potential against breast cancer, spanning the period from 1990 to 2022, has been presented. This review article also outlines the latest trends in endophyte-based drug discovery, including the use of artificial intelligence, machine learning, multi-omics approaches, and high-throughput strategies. The challenges and future prospects associated with fungal endophytes as substitutive sources for developing anticancer drugs targeting breast cancer are also being highlighted.

## 1 Introduction

Breast cancer is a widespread issue with a high global prevalence. Despite adjuvant chemotherapy, the chance of survival for individuals with metastatic breast cancer is below 30 % within the last five years [1]. As per the data provided by International Agency for Research on Cancer (IARC), breast cancer accounts for 2.3 million new cases globally and the fatality rate is recorded as 6.9 % [2]. High-income countries exhibit a greater incidence of breast cancer in comparison to low-income countries, suggesting a correlation with globalization [3,4].Cumulative data reveals that varied subtypes of breast cancer are coupled with differing age groups and ethnicities [[5], [6], [7]]. Younger and premenopausal women, particularly African-American and Asian women, are more commonly affected by triple-negative breast cancer and HER-2 positive subtypes.They have a high rate of relapse [[8], [9], [10], [11], [12]].

Major factors that increase the risk of breast cancer in developed nations include modified lifestyles, late-night work schedules, delayed marriage and first child, and hormone replacement medication [3,13]. The principal reasons for the elevated occurrence and mortality rates of breast cancer in underdeveloped countries are insufficient awareness, inadequate screening programs, late diagnosis, and limited therapeutic resources [4,14].A diverse range of therapies exist for managing breast cancer, encompassing surgical procedures, chemotherapy, radiation therapy, immunotherapy and endotherapy [15,16]. Notwithstanding the existence of these treatments, the incidence and death rates from breast cancer are still rising [17,18]. Effective, targeted treatments with minimal adverse effects are essential for treating breast cancer. Given that breast cancer is a global concern, prioritizing the reduction of disparities in access to diagnosis, multimodal treatments, and innovative medications is imperative.

Contemporary research indicates that natural products may hold a pivotal role in the exploration and advancement of novel pharmaceuticals, owing to their potential to furnish a plethora of distinct and unique templates for future medication [[19], [20], [21], [22]]. With the swiftly evolving insights that a significant proportion of natural products originate from microbial synthesis or microbial interactions with their hosts, the domain of endophyte research for natural products is poised to transform drug discovery and development [23,24]. Endophytes are considered to be distinct microorganisms that internally reside within the tissues of plants, exerting beneficial effects on their hosts, and capable of producing a diverse array of secondary metabolites that possess various biological functions [[25], [26], [27]]. Endophytes establish a spectrum of intricate biological interactions both amongst themselves and with their host plants. However, merely a minute proportion, approximately 0.75–1.50 %, of identified plant species have undergone comprehensive endophyte research, indicating substantial untapped potential for identifying novel bioactive compounds from the extensive array of unexplored plant species [23,28].

This unique group of microbes has drawn attention from researchers due to the discovery of hundreds of diverse chemical classes of metabolites through its isolation, growth, purification, and characterization [29,30]. Endophytes have been found to contain many cytotoxic compounds, including paclitaxel (Taxol) [31], which could be investigated as potential anticancer drugs. Fusarubins produced by *Cladosporium* species [32] and *Fusarium* species [33], including anhydrofusarubin and fusarubin (FUS), have shown promising cytotoxicity against cancer cells. While FUS was previously thought to have antibacterial properties, its cytotoxic activity was recently discovered. The molecular mechanism of the cytotoxic effect of fusarubin from *Cladosporium* species found in *Rauwolfia serpentina* leaves has also been documented [34].

The emergence of these findings has opened up novel avenues for the commercialization of medicinal compounds derived from endophytic fungi. The escalating demand for natural products, coupled with the difficulties associated with obtaining them directly from plants, underscores the allure of endophytes as promising targets for evaluating and extracting host-derived compounds [35,36]. Although medicinal plants are a valuable reservoir of therapeutic compounds, it is imperative to investigate their endophytes as well, to effectively isolate these bioactive compounds. Endophytes are recognized as sustainable, widely available, and environmentally friendly sources of novel bioactive compounds due to their vast diversity and ability to colonize diverse environments [37].

Hence, this comprehensive review highlights the immense potential of fungal endophytes as a valuable reservoir for the development of pioneering anticancer agents for breast cancer therapy.A thorough literature survey from 1990 to 2022 has motivated the authors to elucidate endophytic fungal metabolites associated with medicinal plants as a fascinating group of anticancer compounds depicting promising cytotoxicity against various breast cancer cell lines. This review sheds light on the current state of *in vivo* investigations and clinical trials concerning endophytic fungal metabolites in breast cancer research, providing an overview of their present status. In conclusion, this review discusses the challenges and future directions for exploring these endophytic fungal metabolites in the pursuit of uncovering promising drug candidates to combat breast cancer.

## 2 Current epidemiological statistics of breast cancer

Breast cancer impacted approximately 6.8 million women globally in the year 2018. The information available from cancer registries is limited as it only captures data on the number of cases or deaths from cancer, without accounting for the number of women who have had metastatic cancer and have since become cancer-free [38,39]. Breast cancer occurrence differs globally due to differences in education, economic status, environment, eating habits, lifestyle, and culture [40,41]. By the year 2040, globalization and the growth of economies could lead to an increase in breast cancer rates in both developing countries (by 64 %–95 %) and developed countries (by 32 %–56 %) [40,41].The highest incidence of breast cancer in India was found to be between the ages of 40–49 in urban areas and between 65 and 69 in rural areas [42]. According to a study conducted in northern India, 26 % of people diagnosed with breast cancer were younger than 35 years old [43]. Differences in eating habits such as tobacco use, alcohol consumption, and non-vegetarian diets also contribute to differences in breast cancer prevalence [44].

According to IARC GLOBOCAN 2020 data, breast cancer has the worst incidence and prevalence in 185 countries [40]. The United States was anticipated to witness roughly 280,000 fresh instances of breast cancer and around 40,000 fatalities in the year 2021 [45]. Breast cancer is estimated to affect one in four women and is expected to cause the death of one in eight women [40]. As per the American Cancer Society, the worldwide cancer burden will escalate to 28.4 million cases by 2040, which is approximately 47 % higher than the burden of cancer recorded in 2020 [46]. Breast cancer is more prevalent in older women. In 2018, there were 645,000 instances of breast cancer and 130,000 fatalities in the premenopausal group, while in the postmenopausal group, there were 14 million cases and 490,000 deaths [40]. Nations with high human development index (HDI) record the maximum incidence of breast cancer in both premenopausal (30.6/100,000) and postmenopausal (253.6/100,000) groups, whereas countries with low and medium HDI report the least premenopausal mortality (8.5/100,000) and postmenopausal mortality (53.3/100,000), respectively [47]. Lack of access to early detection and effective treatment remains a major contributor to the high mortality rates associated with breast cancer in developing countries [48].

In India, breast cancer is the cancer type that follows closely after cervical cancer in terms of its rapid increase. According to the National Cancer Registry Program in India, there were 162,468 new instances and 87,090 fatalities attributed to breast cancer in the year 2018 [49,50]. The annual incidence of breast cancer had risen from 0.46 % to 2.56 %, which is higher than that of cervical cancer, indicating a growing trend of breast cancer cases in India. A survey conducted by the Indian Council of Medical Research (ICMR) in New Delhi revealed that the incidence of breast cancer in India almost doubled between 1982 and 2005 [51]. Breast cancer is becoming increasingly prevalent among younger generations in India and has a poorer prognosis compared to Western countries [52]. A local survey found that 52 % of breast cancer patients were between the ages of 40 and 49, and a significant number were under the age of 30 [53]. The proportion of breast cancer patients who survive is relatively low in India due to late-stage diagnosis, with 60 % of women being diagnosed at stage III, while only 1.4 % are diagnosed at stage I [54]. The average tumour size recorded in India is 3.56 cm, with 18.2 % being 2 cm, 65.1 % being 2–5 cm, and 16.7 % being over 5 cm. Of all breast cancer patients in the United States, 64 % have the disease localized, 28 % have regional spread, and 6 % have distant metastasis [50,53].

Socioeconomic factors, education level, marital status, and place of residence can attribute to delayed diagnosis of breast cancer [43]. In India, the incidence rate of breast cancer per 100,000 population varies in diverse cities such as Delhi, Kolkata, Mumbai, Bangalore and Chennai with rates of 41 %, 25.5 %, 33.6 %, 34.4 % and 38 % respectively [55,56]. Among Indian women, TNBC subtype is the most prevalent and destructive form of breast cancer, accounting for 20–43 % of all cases [55]. A study found that the frequency of TNBC is higher in India than in Western countries due to BRCA1 mutations, high mitotic rates, obesity, lifestyle, socioeconomic status, family history etc. [57]. Young indian women also tend to have a high prevalence of the HER-2 positive subtype, while the Luminal A subtype is the least common in this age group [55]. To address this issue, it is vital to adopt a comprehensive approach to breast cancer management that includes raising awareness through campaigns and providing access to medical facilities for women in both urban and rural areas. By doing so, we can work towards reducing the occurrence and mortality rates associated with breast cancer.

## 3 Leads and hurdles in modern breast cancer therapy

When therapy with a curative objective for breast cancer is delivered to completion within a specific timeframe, the outcomes are optimal; its success is dependent on prompt and individualised multimodality treatment referrals following a clear diagnosis [58]. A coordinated and multidisciplinary approach is crucial, with trained healthcare professionals providing essential treatments while minimizing financial and logistical burdens for the patient. Supportive care and pain management are necessary for patients who have been diagnosed with metastatic breast cancer. However, limited resources, inadequate infrastructure, and a shortage of trained staffs may hinder the provision of effective cancer management in low- and middle-income countries. Delays in diagnosis are common in these countries due to a lack of pathology services and the additional costs for tests [59]. These delays can result in a lower chance of survival as more advanced disease stages are often seen in patients with a therapy delay of over three months [60,61].

Widespread screening program for breast cancer that targets the general population in India has led to increased awareness efforts from both government and non-government organizations, highlighting the importance of early detection. The government has improved the quality of care for patients through the provision of a minimum standard of care and additional financial aid for those in need. Although these efforts have been made, advanced breast tumors remain a key concern, particularly in hospitals located in rural and remote areas, where patients frequently turn to alternative therapies before seeking treatment at a cancer centre [62,63]. Nonetheless, there is a reduction in the advanced-stage breast cancer tumors as a growing number of patients undergo neoadjuvant chemotherapy and either axillary clearance or mastectomy [62]. Access to drug therapy has also been improved through the distribution of low-cost generic cytotoxic drugs by the government and non-profit organizations [[64], [65], [66]].Adjuvant radiotherapy is offered to most patients who undergo breast conservation treatment, but many struggle to receive it due to limited infrastructure or poor accessibility [67]. Despite the expansion of radiotherapy facilities, they are still insufficient to meet the demand, resulting in extended waitlists and treatment postponements. However, the adoption of hypofractionation schedules has improved the efficiency of radiotherapy and increased treatment compliance [68].

Treatments for breast cancer such as surgery and chemoradiation in India is only available in major cities, with quality varying based on the treating center, availability of multimodality treatments, and economic background and family support. Delays in diagnosis and treatment lead to poor survival outcomes [69]. In India, there is a delay of 6.1 weeks due to the patient and 24.6 weeks due to the system, leading to a total delay of 29.4 weeks [70]. The reason for low engagement in treatment and follow-up is attributed to the expenses associated with treatment and the social stigma surrounding it [71], with treatment including radiation, surgery, and investigations costing between INR 5–6 lakhs and 6 cycles of customized chemotherapy costing over INR 20 lakhs. The cost of targeted therapy for stage I HER2 + breast cancer, for example, is estimated to be equivalent to 10 years of average annual income in India [72]. With breast cancer treatment becoming increasingly expensive, primary prevention is crucial. Breast cancer has a 100 % success rate when detected early and a high survival rate in the middle stages; however, in India, most of the patients are diagnosed in stage 3 and 4, resulting in low survival rates and elevated treatment costs [73].

Many patients do not receive proper care before being transferred to higher-level medical facilities. Research has shown that between 25 and 40 % of patients in northern India receive local excision or inadequate surgical treatment before being referred [74,75]. Patients who have undergone previous surgery tend to have poor outcomes after being referred. To improve patient care, the Asian Society of Mastology has developed guidelines that involve triple evaluation for diagnosis and recommend breast-conserving surgery (BCS) for individuals diagnosed with early-stage breast cancer whose tumor size measures 4 cm or less [76].Following BCS, adjuvant radiation therapy is administered, and a sentinel lymph node biopsy is conducted on patients who present clinically negative axilla. For breast cancer that is locally advanced, the guidelines suggest neoadjuvant chemotherapy as the initial treatment, followed by a modified radical mastectomy [76].

The fundamental concepts of systemic therapy, comprising cytotoxic chemotherapy, targeted therapy, endocrine therapy and immunotherapy, hold significance in breast cancer treatments, regardless of disease stage and available resources. Nonetheless, the restricted availability of vital oncological medications and treatment services obstructs the application of existing treatment protocols that are imperative for ensuring the secure and efficient delivery of cancer drugs. To mitigate these inequalities, resource-sensitive [77] and resource-stratified guidelines [78,79] have been created to provide general models for interventions in low-resource settings. These guidelines are based on the premise that accurate staging, surgery, and radiation therapy are accessible within the region. However, the practicality of applying treatment guidelines can differ significantly depending on factors such as the number of patients, the distribution of disease stages, the availability of resources, and the level of support provided by the healthcare system [80].

Despite notable strides in anti-HER2 therapy and targeted drugs such as CDK4/6 inhibitors, these treatments are recurrently inaccessible in LMICs. While the median overall survival of HER2-positive metastatic breast cancer patients has improved dramatically in the past 20 years due to HER2 therapies [81], the availability of resource-intensive treatments and accompanying diagnostics required for these therapies is typically limited in LMICs. Likewise, while CDK4/6 inhibitors have been demonstrated to enhance the survival rates of individuals with hormone receptor-positive metastatic breast cancer, these medications are often unavailable to the wider population residing in LMICs [[82], [83], [84]].

Chemotherapy encompasses various categories of cytotoxic medications, namely alkylating agents, antimetabolites, and tubulin inhibitors, as outlined by Ref. [85]. One such alkylating agent is cyclophosphamide, which induces fragmentation of DNA strands, as elucidated by Ref. [86]. Anthracyclines, including doxorubicin, daunorubicin, epirubicin, and idarubicin, exert their mechanism of action through DNA intercalation, thereby impeding the process of macromolecular biosynthesis [87]. Taxanes, such as docetaxel and paclitaxel, operate by binding to microtubules and impeding their disassembly. This action causes cell cycle arrest and triggers apoptosis [88]. Chemotherapy can be administered in different treatment settings for breast cancer, including the neoadjuvant or adjuvant setting, as well as for metastatic breast cancer. The specific administration of chemotherapy may be guided by the molecular subtype of breast cancer, with the inclusion of targeted therapies based on the subtype. Following the completion of chemotherapy, patients diagnosed with hormone receptor-positive breast cancer are recommended to undergo endocrine therapy, while HER2+ breast cancer patients should receive a combination of trastuzumab and chemotherapy [89]. Front-line therapy for patients with TNBC typically involves a combination of taxane and anthracycline [90].

One of the primary limitations of chemotherapy is the occurrence of side effects. During the initial phase of treatment (0–6 months), common side effects include fatigue, alopecia, cytopenia, muscle pain, neurocognitive dysfunction, and chemotherapy-induced peripheral neuropathy. In the later stages of treatment (after 6 months), chemotherapy can give rise to chronic or long-term side effects, including cardiomyopathy, the development of secondary cancers, early onset of menopause, infertility, and psychosocial impacts [91]. Moreover, each of these cytotoxic drugs has the potential to induce resistance in breast cancer patients [92]. An established mechanism of resistance involves the overexpression of p-glycoprotein, a member of the ATP-binding cassette (ABC) family. This overexpression of p-glycoprotein leads to resistance against anthracycline and taxane medications, impairing their effectiveness in breast cancer treatment [93]. The overexpression of Breast Cancer Resistance Protein (BCRP), another member of ABC family, has been found to induce resistance specifically to anthracycline medications but not taxanes [94].

Alterations in microtubules can contribute to taxane resistance. The overexpression of β-tubulin III, a specific isoform of tubulin protein, has been shown to induce resistance to paclitaxel, thereby reducing its efficacy in treating breast cancer [95]. Furthermore, mutations in microtubule-associated proteins (MAPs) can impact microtubule dynamics and enhance resistance to taxanes [96]. Surgery, radiotherapy, and chemotherapy are integral components of breast cancer treatment, but they may not be adequate for effectively treating all molecular subtypes of breast cancer due to variations in their response to radiotherapy and chemotherapy. Gaining insight into the mechanisms of drug resistance is crucial for the development of novel breast cancer treatments. Targeting the mTOR/PI3K/Akt pathway, which is involved in resistance across all molecular subtypes, shows promise as an effective approach for treating breast cancer [97].

Certainly, there is an urgent necessity for novel, safe, and less hazardous therapeutic options derived from natural products to tackle this escalating health crisis. Noteworthy progress has been made in cancer therapy over the past few years, primarily due to the exploration and refinement of chemotherapeutic drugs sourced from natural products [98,99]. Bioactive compounds produced by living organisms, comprising of plants, animals, and microbes, are known as natural products.These compounds, commonly referred to as secondary metabolites, are distinct from primary metabolites as they are not crucial for an organism's growth or reproduction.Instead, these secondary metabolites may be synthesized as a response to ecological adaptations or as a means of deterring predators, which enhances the organism's likelihood of survival [100]. They are often characterized by their small organic structures and wide-ranging biological activities [101], that have played a pivotal role in modern medicine. This is evident by the fact that nearly 42 % of recently permitted pharmaceuticals are either natural products or their derivatives, and over 52 % of small molecule drugs authorized for anti-infective use during the same timeframe have roots in natural origins [[102], [103], [104]].

Phytochemicals have anticancer properties, and many of them are currently being utilized in cancer therapy [[105], [106], [107]]. However, despite being considered the most promising source of potential medications, phytochemicals have faced a decline in attention in drug research due to various challenges associated with using plants as a source of drug molecules, such as their unique habitats, slow growth rates, limited yields, difficulty in reproducing desired phytochemicals, and ongoing threats from human activities [108,109]. In contrast, microorganisms have garnered greater interest as a possible source of pharmaceutical agents due to their widespread distribution, high diversity and ability to produce unique bioactive compounds. Microbes can be easily cultured in controlled environments and consistently produce the desired chemicals, making them an attractive source for drug discovery and development [24]. The increasing awareness that a notable fraction of natural products are synthesized by microbes or result from the interactions between microbes and their hosts has opened up avenues for investigating endophytes in the quest for discovering natural products [23,24]. In 1993, the revelation that the chemotherapeutic drug Paclitaxel, previously believed to be sourced from the plant *Taxus longifolia*, was actually synthesized by endophytic *Taxomyces andreanae* from the same plant, opened up the possibility of plants being a valuable reservoir of microbial diversity, or endophytes [110].

The fatality rate of breast cancer is significantly influenced by the stage at which the disease is diagnosed, underscoring the critical role of early detection in improving patient outcomes. The earlier breast cancer is detected, the more treatment options are available, generally leading to better prognoses and lower fatality rates. This is crucial because the survival rates for early-stage breast cancer are significantly higher than for later stages. For instance, localized breast cancers (those that have not spread beyond the breast) have a 5-year survival rate of nearly 99 %. Conversely, later-stage breast cancer, which may be detected without screening or in populations without adequate screening programs, has a poorer prognosis and a higher fatality rate. Advanced-stage breast cancer survival rates drop significantly, illustrating the lethal impact of delayed diagnosis. Recent research has begun exploring how fungal endophytes might contribute to cancer therapy, including breast cancer. Endophytic fungi are microorganisms that live inside plant tissues without causing apparent harm to their host. They are known for producing a plethora of secondary metabolites, many of which have been found to possess bioactive properties, including anticancer activities. Their role primarily lies in the potential development of new therapeutic agents that could be used either alone or in conjunction with existing treatments to manage breast cancer more effectively. By potentially offering new treatments, these fungi could be part of a broader strategy that includes early detection to improve patient outcomes. Early and effective treatment following detection reduces the fatality rate by controlling the disease before it progresses too far. While early detection directly influences the fatality rate of breast cancer by facilitating early and effective treatment, endophytic fungi contribute indirectly by offering new avenues for therapeutic intervention. Their exploration and integration into cancer therapy hold promise for enhancing treatment efficacy, which is crucial once cancer has been detected. Hence, by acknowledging these valuable insights, the current review would aid in broadening the future search and discovery of numerous novel bioactive compounds with the potential for breast cancer therapeutics and drug development.

## 4 Endophytic bioactive metabolites as a treasure trove of novel anticancer agents

Endophytes are microorganisms that dwell within the tissues of plants in a mutually beneficial relationship, without causing any observable signs of disease [111]. Endophytes can produce bioactive compounds that resemble those synthesized by the host plant during mutualistic interactions [112].For example, anticancer drugs such as podophyllotoxin, camptothecin, taxol and vincristine have been synthesized by endophytic fungi isolated from *Podophyllum hexandrum*, *Camptotheca acuminata*, *Taxus chinensis* var. mairei and *Catharanthus roseus* [[113], [114], [115], [116]].Furthermore, these endophytes are capable of producing unique secondary metabolites that possess novel structures [117],and in certain cases, hybrid chemical scaffolds [118].

The discovery of unique and innovative structures in endophytic fungal metabolites has contributed to the development of novel drugs. Most of the small molecule drugs that have been approved or considered acceptable are either natural compounds, synthetic compounds that mimic the biological functions of natural compounds, or synthetic analogues of natural compounds [119]. They play a crucial role in drug discovery due to their structural variety and physiological activity [120]. Endophytes produce biomolecules that enhance ecological adaptability and co-evolve with their host plants, resulting in the production of functional natural compounds. As an example, the endophytic fungus *Neotyphodium coenophialum* discovered in *Festuca arundinacea* produces poisonous alkaloids that aid the host in deterring herbivores by inducing "fescue toxicosis" [121].These alkaloids have potential medicinal value in treating various diseases.

The genera *Aspergillus, Penicillium, Chaetomium, Pestalotiopsis* and *Fusarium* are among the most frequently identified endophytic fungal species. Extensive global research has been conducted on the diversity and abundance of endophytic fungi, with the aim of harnessing their valuable resources, as numerous studies have demonstrated that they represent a promising source of bioactive compounds [[122], [123], [124], [125]]. Various chemical groups have been identified to classify endophytic fungal metabolites, including but not limited to alkaloids, terpenoids, steroids, lactones, polyketides, flavonoids, lignans, quinones, depsipeptides and others.Over the course of the last 30 years, more than 180 natural compounds that exhibit noteworthy cytotoxicity against specific tumor cell lines have been identified from endophytic fungi [122,124].

Torreyanic acid is a bioactive compound renowned for its anticancer properties which was found to be synthesized by the endophytic fungus *Pestalotiopsis microspora* inhabiting *Taxus taxifolia*. The ability of torreyanic acid to induce apoptosis in cancer cells is well-documented, especially in cancer cell types that are sensitive to protein kinase C agonists [126].Another group of anticancer agents are cytochalasins, which are alkaloids synthesized by about 20 species of endophytic fungal genera such as *Chalara, Hypoxylon*, *Xylaria* and *Phoma* [127].Camptothecin, another potent antineoplastic agent, has been discovered in various endophytic fungi, including *Entrophospora infrequens*isolated from*Nothapodytes foetida* [128].In addition, topotecan and irinotecan, which are similar in structure and function to camptothecin, were discovered from *Fusarium solani*inhabiting*Camptotheca acuminate*. The potential of these compounds as viable and effective anticancer drugs has been demonstrated in clinical studies [129].Endophytic fungi *Phomopsis vaccinia, Colletotrichum gloeosporioides, Alternaria alternate* and *Fusarium nematophilum*, all recovered from *Cyanea acuminate* [130] have also been found to produce camptothecin.

*Trametes hirsute*, an endophytic fungus, produces a nonalkaloid lignin called podophyllotoxin, which has been found to have significant anticancer potential [131]. Other endophytic fungal species, including *Aspergillus fumigates* from *Juniperus communis* [132], *Fusarium oxysporum* from *Juniperus recurva* [133] and *Phialocephala fortinii* from *Podophyllum peltatum* [134] have also been found to produce podophyllotoxin and its analogues, indicating that this compound is not unique to *Trametes hirsute*. Endophytic *Podophyllum hexandrum* has been found to produce cathartic, emetic, and cholagogue lignans that also exhibit anticancer effects. Resins from *P. emodi*, including etoposide and teniposide, have also been discovered to possess significant anticancer potential [135]. Huperzine A, a compound with cholinesterase inhibitor properties, was found to be produced by *Huperzia serrata* [136]. Additionally, the endophyte *Mycelia sterilia*, which was found to be associated with *Catharanthus roseus*, produced vincristine, a potent bioactive compound used primarily as a chemotherapeutic drug for nephroblastoma and acute lymphoblastic leukemia [137].

The field of endophyte characterization holds great promise due to the discovery that medicinal plants harbor diverse microorganisms that synthesize bioactive compounds within their tissues and organs [138]. Therefore, medicinal plants research can provide valuable insights into their potential use as alternative sources of medication, including endophytes. By tapping into medicinal plants' endophytic communities, we can discover unidentified or novel bioactive metabolites with significant pharmacological value [139]. At present, research endeavors are underway to comprehensively comprehend the structural variety and novel functional properties of medicinally potent endophytic compounds derived from plants, with the objective of therapeutic applications.

## 5 Exploring the diversity of endophytic fungi residing in medicinal plants

The copious presence of fungal endophytes in medicinal plants is widely recognized for their capacity to produce a wide range of bioactive metabolites with promising therapeutic potential [140]. Various phyla of fungi, including Basidiomycota, Mucoromycota, Oomycota and Ascomycota, have been identified as endophytes in diverse plant species. Among these phyla, Ascomycota is home to more than half of all known endophytic fungi, followed by Basidiomycota, while Oomycota has the smallest number of known fungal species. As per a study, the most prevalent genera of endophytic fungi found in plants are *Aspergillus, Fusarium, Penicillium*, and *Piriformospora* [141]. Additionally, specific fungal strains adapted to particular niches have also been identified. For example, *Penicillium glabrum* and *Penicillium brevicompactum* were found in barley (*Hordeum vulgare*), while *Cryptococcus, Gibberella zeae*, *Berkleasmium* and *Chaetomium*were derived from maize (*Zea mays*). Furthermore, *Gibberella moniliformis, Didymella bryoniae*, *Diaporthe helianthi, Diaporthe phaseolorum, Guignardia vaccinii* and *Leptospora rubella*were isolated from soybean wheat (*Triticum aestivum*) [142,143].

Endophytic fungi have been discovered in a multitude of medicinal plants, and have been found to synthesize economically valuable bioactive compounds [144]. conducted a study which demonstrated a higher occurrence of *Pestalotiopsis* sp. and *Penicillium* in mature leaves and petioles of the indigenous plant *Cordemoyaintegrifolia*, in comparison to younger leaves. Thai medicinal plants have been found to contain diverse fungal endophytes that can produce bioactive compounds, such as the anticancer agent Camptothecin [128]. *Entrophospora infrequens*, found in the inner bark of *Nothapodytes foetida*, was identified as the source of Camptothecin.

[145] reported the presence of endophytic fungi from leaves and branches of five *Garcinia* species [146]. reported the discovery of *Penicillium thomi* in the roots of *Bruguiera gymnorhiza*, and this endophytic fungus was utilized to produce a novel molecule named 4′, 5 dihydroxy-2,3 dimethoxy 4(-hydroxy propyl)-biphenyl, which was assessed for its cytotoxic effects on three human cell lines [147]. identified 1160 endophytic fungi from 29 Chinese medicinal plants, with the most commonly isolated genera being *Phoma*, *Colletotrichum*, *Xylariales* and *Phomopsis* [148]. discovered Taxol, an important anticancer agent, synthesized by the fungus *Bartalinia robillardoides*, which inhabits *Aegle marmelos*.

An investigation into the endophytic fungi present in *Monarda citriodora* Cerv. ex Lag was conducted [149].Their investigation uncovered a total of 28 fungal endophytes, which were classified into the phylum Ascomycota comprising 11 discrete genera. Results showed that roots of the plant exhibited the highest fungal dominance in terms of tissue specificity, while leaves displayed a wide variety of fungal species. Additionally, the researchers found that extracts from 72 % of the isolated endophytic fungi displayed cytotoxic effects onmultiple types of human cancer cells. Remarkably, significant anticancer activity was observed in the extracts obtained from *Aspergillus fumigatus* (MC-18 L), *Cladosporium tenuissimum* (MC-24 L), *Fusarium oxysporum* (MC-14 L, MC-14 F, and MC-26 F), and *Fusarium* sp. (MC-25 L), as they exhibited IC_50_ values of 10 μg/mL or lower. Another investigation involved the isolation of 154 fungal endophytes from the roots and stems of *Distylium chinense* [150].

*Withania somnifera* (L.) Dunal, is a valuable medicinal herb with a range of therapeutic properties, widely used in Ayurvedic medicine. Abundant fungal endophytes have been isolated from different tissues of *W. somnifera* that were gathered from Kerala, Madhya Pradesh, Western Himalayas, and Pakistan [151]. Specific endophytes in association with *W. somnifera* can enhance the biosynthesis and accumulation of specific secondary metabolites [152].A group of 11 fungal strains capable of enhancing the growth and secondary metabolite production of *W. Somnifera*were isolated and identified [153].*Sarocladium implicatum, Penicillium* sp., *Trametes versicolor* and *Aspergillus terreus*were among the fungal isolates recovered from the leaves, while *Colletotrichum capsici, Penicillium oxalatum, Ceratobasidium* sp., *Aspergillus brasiliensis, Penicillium* sp., *Hypocrea lixii* and *Colletotrichum truncatum* were among the fungal isolates recovered from the roots. Endophytic fungi from *W. somnifera*, encompassing 21 genera and 38 species were isolated and identified, with *Cladosporium, Alternaria, Setosphaeria* and *Aspergillus* being the most predominant ones [154].

Turmeric, a member of the Ginger family, is widely used in Ayurvedic medicine to treat a variety of ailments. Prana et al. have recovered 45 uncharacterised fungal endophytic isolates from the rhizomes of the turmeric plant [155], while [156] have identified *Phyllosticta* sp. from *Panaxquinquefolium*, and [157] have identified *Aspergillusclavatus* from *Azadirachta indica*. 41 endophytic fungi were identified from yellow vine, an endangered medicinal herb, with *Phomopsis jacquiniana* being the predominant fungus [158]. Furthermore [159], isolated 53 fungal endophytes from the roots and stems of *Dendrobium thyrsiflorum* and *D. devonianum* with *Fusarium* sp. being the most abundant genus. Additionally [160], succeeded in isolating a total of six endophytic fungal strains from *Albizia* plants, comprising *Acremonium* sp., *Fusarium* sp., *Aspergillus* sp., *Trichoderma* sp., *Verticillium* sp. and *Penicillium* sp. Therefore, investigations keen on the vast communities of endophytic fungi found within medicinal plants may ultimately contribute to the discovery of highly effective therapeutic compounds for breast cancer therapy.

## 6 Efficacious anticancer compounds synthesized by endophytic fungi against breast cancer

Chemotherapeutic drugs have been a longstanding treatment for cancer, but they often come with harmful effects on cells. To mitigate these concerns, natural anticancer compounds sourced from endophytic fungi have emerged as an alternative therapy for a wide range of cancers, including breast cancer, lung, ovarian, prostate and Kaposi's sarcoma. These compounds have demonstrated the ability to stimulate apoptosis and impede cancer progression [161,162].This section delves into the intricacies of several endophytic fungi-derived anticancer drugs and their mechanisms of action against breast cancer.

## 7 Paclitaxel (Taxol)

**Fig. 1:**
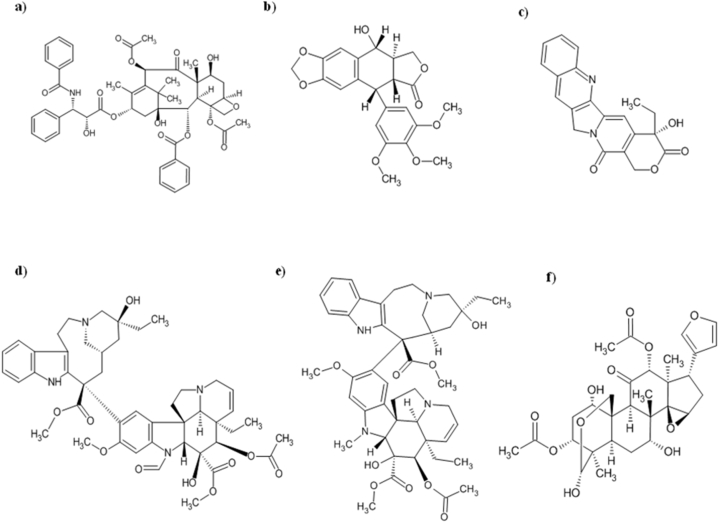
Endophytic fungi derived anticancer compounds against breast cancer: a) Paclitaxel; b) Podophyllotoxin; c) Camptothecin; d) Vincristine; e) Vinblastin; f) Toosendanins.

Taxol, an anticancer drug that generated over a billion dollars in revenue, belongs to the taxanes class of highly functionalized polycyclic diterpenoids. Researchers have been actively probing for alternative sources of paclitaxel due to the low yields of taxol derived from plants.Identifying endophytic fungi as taxol producers resulted from the search for substitutes (Fig. 1a) [163].The discovery of taxol-producing endophytes in both *Taxus* and other plants prompted an investigation into diverse plant species, aimed at uncovering further taxol-producing endophytes. Several genera of endophytic fungi, including *Alternaria, Botryodiplodia Aspergillus, Botrytis, Ectostroma, Metarhizium, Mucor, Phyllosticta, Taxomyces*, *Monochaetia, Pestalotia, Pestalotiopsis, Fusarium, Periconia, Cladosporium, Ozonium, Pithomyces,* and *Papulaspora* have undergone evaluation and have been found to produce paclitaxel and its analogues [31,[164], [165], [166], [167], [168], [169], [170], [171]].

Numerous investigations have examined the cytotoxic properties of taxol and its precursors against breast cancer as a means to explore their potential as anticancer agents. One such intriguing study found that a biosynthetic precursor of taxol known as baccatin III, has a comparable mode of action as taxol. The inhibitory impacts of Taxol and baccatin III extracts derived from *Fusarium solani* were assessed on T47D breast cancer cells. In T47D cells, both compounds were observed to hinder cell proliferation, with taxol exhibiting IC50 values ranging from 0.005 to 0.2 M, and baccatin III demonstrating values between 2 and 5 M. This research indicated that, while both fungal taxol and baccatin III treatments induce apoptosis in the cells tested, there are differences in the susceptibility of tumour cells to these compounds. The matter of which compound is the superior inducer of apoptosis is still a topic of discussion, as baccatin III has shown to be more effective than taxol inside cells during the growth phase, but less effective than taxol in *in vitro* experiments. Taxol outperforms baccatin III when it comes to cellular absorption, microtubule binding kinetics, and protein interactions [172]. [173] extracted taxol from *Colletotrichum capsici* associated with damaged chilli fruits and showed that it has potent anticancer effects on MCF-7 cells at various doses namely 0.005, 0.05, 0.5, and 5 μM respectively. The study observed that the taxol produced by the fungal endophyte exhibited the most potent anticancer effect against MCF-7 cells at 0.5 μM with 79.37 (±7.57) percentage of apoptotic cells, surpassing its efficacy at other doses. The decrease in cell proliferation was achieved through a process involving both apoptosis induction and mitosis inhibition. Specifically, apoptosis induction preceded mitosis inhibition, resulting in the observed decrease in cell proliferation.

Paclitaxel's efficacy as an anti-cancer medication can be ascribed to its capacity to trigger cytotoxicity at various points throughout the cell cycle. It has been found to influence the formation and maintenance of microtubules, specifically by stabilizing microtubules during polymerization and preventing depolymerization, which in turn disrupts microtubule assembly. Studies have shown that paclitaxel, when combined with calcium chloride (4 mM), can resist or delay depolymerization which causes instability of microtubules, by interfering with the spindle network. Besides its cytotoxic effects, paclitaxel is also recognized for its crucial role in cell signaling pathways. It does so by interrupting the microtubule network, which then causes increased levels of Bax and Bcl-2. As a consequence of this interference, the cell cycle is halted in the G2-M phase, and subsequent cell death occurs as a result of apoptosis during the G1 phase of mitosis. These effects have been demonstrated in various studies [[174], [175], [176]].

## 8 Podophyllotoxin

Podophyllotoxin, an aryl tetralin lignan, has been studied for its potential as an anticancer agent due to its cytotoxic and antiviral properties (Fig. 1b) [177]. Its analogues are also of pharmacological interest [[178], [179], [180]]. Etoposide and teniposide, which are semi-synthetic derivatives of podophyllotoxin, have been approved for the treatment of various cancer types, such as testicular and lung cancers, solid tumors, and leukemia [129]. In 1966, etoposide was created as a substitute for podophyllotoxin and subsequently gained FDA approval in 1983.

Two strains of *Phialocephala fortinii* were isolated from the rhizomes of *Podophyllum peltatum* which exhibited the ability to synthesize podophyllotoxin in quantities varying between 0.5 and 189 μg/L. After conducting a brine shrimp lethality assay, the cytotoxicity of the fungal extract was evaluated, revealing LD50 values ranging from 2 to 3 μg/mL [134]. Likewise, in India's Gulmarg region, *Fusarium oxysporum* inhabiting *Juniperus recurva*, was discovered to generate podophyllotoxin at a rate of 28 μg/g dry mass [133]. *Trametes hirsuta,* an additional endophytic fungus, was obtained from desiccated rhizomes of *Podophyllum hexandrum* and was determined to synthesize podophyllotoxin, demethoxypodophyllotoxin and podophyllotoxin glycoside. The resulting compounds displayed anticancer activity towards U-87 cell line [131].

During a research investigation, the presence of *Aspergillus fumigatus* Fresenius was detected in *Juniperus communis* L. Horstmann specimens obtained from the Rombergpark botanical gardens in Dortmund, Germany. This fungal strain was able to biosynthesize deoxypodophyllotoxin, with an optimal yield of 100 μg/g of mycelial dry weight [129]. During another research study, the rhizomes of *Sinopodophyllum hexandrum* (Royle) Ying, a plant species found in the Taibai Mountains of China, were found to harbor six endophytic fungi. Within this collection of fungi, *Mucor fragilis* Fresen was discovered to synthesize podophyllotoxin and kaempferol, yielding 49.3 μg/g of mycelial dry weight [181]. In a recent development, the biosynthesis of podophyllotoxin from *Alternaria tenuissima* was reported for the first time. *Alternaria tenuissima*was identified in the collected roots of *Sinopodophyllum emodi* (Wall.) Ying, procured from the Xinglong Mountains located in China's Gansu Province. Secondary metabolite analysis was used to detect the presence of podophyllotoxin in the fungal biomass [182].

Studies have demonstrated that podophyllotoxin and its analogues have the potential to be effective targeted therapies that can specifically target and efficiently impede the growth, movement, and invasion of breast cancer cells in both *in vitro* and *in vivo*settings [183]. For example, acetylpodophyllotoxin exhibited selective inhibition towards BT-549 cells [184] and podophyllotoxin piperazine acetate derivatives caused G2/M blockage and microtubule disruption in MCF-7 cell line while minimizing harm to noncancerous cells [185]. In MCF-7 cells, 4β-amidopodophyllotoxins led to cell cycle arrest, elevated expression of p53 and cyclin B1 proteins, and reduced Cdk, indicating interference with mitosis [186]. Additionally, treatment of MCF-7 cells with these podophyllotoxin derivatives resulted in suppression of protein expression such as VEGF-A, STAT-3, ERK1/2, ERK-p that regulates the tumour microenvironment suggesting the potential for influencing tumour angiogenesis and invasion.

Podophyllotoxin exerts its significant cytotoxic effects by interacting with topoisomerase II and inhibiting DNA replication. This mechanism is achieved by increasing the levels of topoisomerase II, which is highly vulnerable to the toxic effects of etoposide. This binding leads to DNA duplex damage, where the double-stranded DNA of mammalian tissues break, thereby increasing the likelihood of disruption in the DNA's integrity. The buildup of DNA damage leads to cell death by causing changes in the DNA structure due to insertions, deletions, and genetic recombination. This mechanism has been extensively studied and reported in published papers [[187], [188], [189], [190]].

## 9 Camptothecin

The bark of *Camptotheca acuminata* was the source of the original discovery of Camptothecin, which is a highly effective anti-cancer agent. This pentacyclic pyrroloquinoline alkaloid has been the precursor for the development of several other anti-cancer medications including irinotecan and topotecan. The natural form of camptothecin is the 20-S-camptothecin enantiomer, while the 20-R-camptothecin enantiomer is biologically inactive [25]. Efforts are being made to discover alternative sources of camptothecin, as the current production from plant sources falls short of global demand. Due to inadequate supply from plant sources, the endophytic fungus *Entrophospora infrequens*, which was obtained from the inner bark of *Nothapodytes foetida* in the Konkan ghats region of India, has been identified as a potential source of camptothecin (Fig. 1c). The fungal camptothecin has demonstrated significant cytotoxicity against MCF-7, A549, HEp2, and OVCAR-5 cells, yielding 18 μg/mg of chloroform extract. The cultivation of the same fungus in a bioreactor for 48 h resulted in the production of 4.96 mg of camptothecin per 100 g of dry mass [128,191].

After four days of incubation in broth culture, two strains of *F. solani* which were isolated from *Apodytes dimidiata*, produced 37 and 53 μg/100 g of camptothecin [192]. Another study reported the isolation of *Phomopsis* sp., *Fomitopsis* sp. and *A. alternata* from *Miquelia dentata*, which were found to synthesize 10-hydroxycamptothecin, 9-methoxycamptothecin and camptothecin. 42.06, 55.49 and 73.9 μg/g dry weight were the yield obtained for these compounds [193].Three endophytic fungi that produce camptothecin, namely *Aspergillus* sp. LY355, *Trichoderma atroviride* LY357, and *Aspergillus* sp. LY341 were extracted from *C. acuminata.* These fungi were found to produce 42.92, 197.82 and 7.93, μg/L of camptothecin, respectively. A study reported the isolation of 161 fungi from *Cola acuminata*, out of which *Botryosphaeria dothidea* X-4 was identified as a producer of 9-methoxycamptothecin [194].In addition, *Neurospora* sp., obtained from *Nothapodytes foetida*, was capable of producing camptothecin and demonstrated notable cytotoxic effects against MCF 7 and OVCAR-5 cells [195].

In patients with endocrine-resistant breast cancer, camptothecin and its derivatives are commonly administered as a second or third-line treatment. In the late 1980s, it was believed that camptothecins worked by blocking DNA and RNA synthesis in cancer cells, but later research revealed that camptothecin targets topoisomerase-I, which is responsible for unwinding DNA supercoiling that occurs during replication. Topoisomerase-I creates nicks in the single strand of DNA to relieve DNA supercoiling, resulting in the formation of topoisomerase-I cleavage complexes. The process involves catalyzing the formation of an ester bond between the catalytic tyrosine and the 3′ end of the nicked DNA. The 5′ hydroxyl group of the nicked DNA strand generated in the process initiates a nucleophilic attack on the tyrosyl-DNA-phosphodiester bond, resulting in the DNA being returned to its original conformation. The detection of topoisomerase-I cleavage complexes in the cellular milieu is typically challenging due to their transient nature [196].Nevertheless, camptothecin and its derivatives can bind and stabilize these complexes, triggering a series of apoptotic responses that eventually culminate in cell death.Importantly, camptothecin does not exhibit any off-target effects, as it was found to be entirely resistant to mutant yeasts (*Schizosaccharomyces pombe* and *Saccharomyces cerevisiae*) lacking topoisomerase-I activity [197].In summary, camptothecin's anticancer properties have been definitively attributed to its capacity to inhibit DNA topoisomerase-I. This enzyme serves a crucial function in regulating DNA supercoiling and relaxationduring critical biological processes such as replication and transcription.

Researchers have conducted small, non-randomized trials for breast cancer and noted a significant range in response rates to camptothecin, ranging from 14 % to 64 %. This variability can be attributed to the lack of dependable selection criteria for effectively categorizing patients.To tackle this challenge, breast cancer cell lines can be employed as effective models to establish functional parameters that can aid in clinical diagnosis and predict drug sensitivity.This approach can help to ensure more appropriate usage of camptothecin in clinical trials.

## 10 Vinca alkaloids

Vinca alkaloids are a specific class of terpenoid indole compounds that arise from the amalgamation of vindoline and catharanthine monomer.Vinca alkaloids have become a frequently used element of chemotherapy treatments due to their ability to lower the number of white blood cells in acute lymphoblastic leukemia and nephroblastoma. Being the most prevalent category of anticancer medications, vinca alkaloids are utilized in the management of a diverse range of cancers [198].The roots of vinca alkaloids' discovery can be traced to the late 1950s, when scientists started investigating the extract of the Madagascar periwinkle plant, which is also referred to as *Vinca rosea* or *Catharanthus roseus* G. Don.After more than a decade of phytochemical research, alkaloids such as vinblastine, vincristine, vinleunosine, and vinrosidine were identified as potent anticancer agents [[199], [200], [201]].

Endophytic fungi inhabiting *Catharanthus roseus* have been found to generate vincristine and vinblastine, two important anticancer alkaloids (Fig. 1d and e). *Alternaria* sp. was discovered to produce vinblastine, while *F. oxysporum* an endophytic fungus from *C. roseus* leaves were found to synthesize vincristine [202,203].Additionally, *F. solani* was observed to be capable of producing both vincristine and vinblastine with production rate of 67 μg/L and 76 μg/L respectively [204,205].Another endophytic fungus originating from *C. roseus, Talaromyces radicus,* was able to produce70 μg/L of vinblastine and 670 μg/L of vincristine.In a dose-dependent manner, vincristine was discovered to inhibit the cell proliferation of several cancer cell lines, including MCF7, A549, HeLa, U251, and A431 cells.The respective IC_50_ values for these cells were 4.5 g/mL, 5.5 g/mL, 4.2 g/mL, 5.5 g/mL, and 5.8 g/mL.Conversely, normal cells (HEK293) did not show any significant impact [113].

In MCF-7, CHO–K1, and HepG-2 cell lines, the growth of cells was significantly suppressed by fungal vinblastine, which had IC50 values of 8.55 g/mL, 12.15 g/mL, and 7.48 g/mL, respectively [206].Moreover, the study found that combining vinblastine (150 nM) and indibulin (50 nM) resulted in a significant suppression of MCF-7 cell proliferation. The combination led to a 71 % and 53 % reduction in proliferation, with a combination index (CI) of 0.5 and 0.67 respectively. A synergistic effect of indibulin and vinblastine on the growth of MCF-7 cells was evidenced by a combination index of ≤1. This finding suggests that the two drugs acted cooperatively to inhibit cell proliferation [207].

Vinca alkaloids function by impeding the functions of microtubule cytoskeleton, thereby targeting cell cycle progression. Microtubules are an essential component of the cytoskeleton, which consist of tubulin heterodimers involved in intracellular cargo transport, cell motility, and cell cycle progression [208].The microtubule's structural arrangement involves the alternating α and β tubulins that assemble into a protofilament. A polar orientation is created by the arrangement that positions the α subunits toward the minus end and the β subunits toward the plus end.A cylindrical structure is formed when the protofilaments align themselves in parallel to create microtubules [209,210].The quick expansion and contraction of microtubules, which gives them their dynamic nature, is a result of the ongoing addition and removal of tubulin subunits. This phenomenon is often called dynamic instability.The maintenance of microtubule length at a steady state is achieved by the addition of free subunits at the plus end and their release at the minus end [211].Numerous methods are employed to impede cell division by interfering with the stability and dynamics of microtubules.

By binding to the vinca domain of β tubulin, which is located near the plus end of the microtubule, Vinca alkaloids disrupt the structure of microtubules.The inhibition of dynamic instability at the plus ends of microtubules [212] by vinblastine results in the prevention of cell division at mitotic metaphase [213].Vinca derivatives exert their cytotoxic effects by binding to intracellular tubulin, resulting in the disintegration of microtubules and the prevention of spindle assembly during mitosis. This mechanism ultimately halts cell division [213,214].These compounds principally affect microtubule assembly, mitotic spindle dynamics, and intracellular transport, exert antineoplastic activity making them potent microtubule destabilizers. Vinblastine and vincristine have been used to treat Hodgkin lymphoma, while other structural analogues (Vinflunine, Vinorelbine, and Anhydrovinblastine) are being investigated in phase-II/III trials for breast cancer therapy by targeting tubulin polymerization [215].

## 11 Toosendanins

Toosendanin (TSN) has been extensively studied in scientific research and clinical medicine, owing to its unique biological properties (Fig. 1f) [216].Preclinical studies conducted in *in vitro* and *in vivo* conditions have confirmed that TSN has potential anti-cancer properties against a variety of cancer types [217], including breast cancer, colorectal cancer, leukemia, prostate cancer, lymphoma, and hepatocellular carcinoma by suppressing the MEK/ERK, MAPK/JNK andPI3K/AKT pathways, which lead to apoptosis and cell cycle arrest [218]. TSN and isotoosendanin (ITSN) had cytotoxic effects on a broad range of tumour cells, with a greater effectiveness observed in TNBC such as BT549, MDA-MB-231 and 4T1 [219]. According to the study, the researchers observed that the treatment with ITSN (2.5 nM) or TSN (20 nM) brought about apoptosis in 4T1 and MDA-MB-231 cells, by decreasing the levels of pro-caspase-3 and Bcl-xL. The natural compounds TSN and ITSN were also discovered to inhibit the growth of TNBCby inducing necrosis and autophagy. These findings demonstrate endophytic fungi as a promising resource of anticancer compoundsand highlight the importance of studying natural sources for drug discovery. In addition to these bioactive compounds, the current review elucidates a list of anti-breast cancer compounds derived from endophytic fungi in (Table 1), which were identified from 1990 to 2022.

**Table 1:** Anti breast cancer compounds isolated from medicinal plant associated endophytic fungi discovered from 1990 to 2022.

Sr. no.	Bioactive compounds	Endophytic Fungi	Medicinal Plant	IC50 (μM) against breast cancer cell lines	References
1.	**Fusarithioamide B**	*Fusarium solani*	*Anvillea arcinia* (Burm.f.) DC.	0.21	[220]
2.	**Spiciferone F**	*Phoma betae*	*Kalidium foliatum* (Pall.) Moq	7.73 ± 0.11	[221]
3.	**Colletotricone A**	*Colletotrichum gloeosporioides* A12	*Aquilaria sinensis*	15.7	[222]
4.	**Flavipin**	*Chaetomium globosum*	*Couroupita guianensis* Aubl. leaves	54	[223]
5.	**Bellidisin D.**	*Phoma betae*	*Tricyrtis maculate* leaves	8.40	[224]
6.	**Demethylchaetocochin C, dethiotetra(methylthio)chetomin, chaetoperazine A, 4-formyl-N-(30- hydroxypyridin-20-yl) benzamide**	*Chaetomium globosum* 7951	*Panax notoginseng* root	4.5 to 65	[225]
7.	**Chetoseminudin F (1), chaetocochin C (6), ergosterol (8), chetomin A (9), chetomin (12)**	*Chaetomium* spp. SYP-F7950	*Panax notoginseng* Stem	(1)- 26.49; (6), (8), (9), (12)- 2. 75 to 8.68	[226]
8.	**Ascomylactam A to C (1**–**3)**	*Didymella* spp. CYSK-4	*Pluchea indica* healthy branch	(1, 3)- 4.2 to 7.8 (2)- 6.6	[227]
9.	**Pleosporalin F**	*Pleosporales* spp. F46	*Mahonia fortunei*	22.4 ± 1.1	[228]
10.	**Sporulosaldein F**	*Paraphaeosphaeria* spp. F03	*Paepalanthus planifolius* leaves	34.4	[229]
11.	**Trichodermic acid**	*Penicillium citrinum*	*Taxus media* roots	42.85	[230]
12.	**Stemphyperylenol**	*Alternaria alternata*	*Psidium littorale Raddi* leaves	4.2 ± 0.6	[231]
13.	**Aspergisocoumrins A & B**	*Aspergillus* spp. HN15-5D	*Acanthus ilicifolius* fresh leaves	(A) 5.08 ± 0.88 and (B) 4.98 ± 0.74	[232]
14.	**Macrophin**	*Phoma betae*	*Glycyrrhiza glabra* Linn	14.8, 8.12, 13.0	[233]
15.	**dechloromaldoxin (1) and 20 - aminodechlorogeodoxin (2)**	*Pestalotiopsis fici.*	*Cinnamomum camphora* branches	(1)- 23.53; (2)- 20.79	[234]
16.	**Stachybochartins A, B, C, D and G.**	*Stachybotrys* sp. PT2–12	*Pinellia ternata*	4.5 to 21.7	[235]
17.	**Peniquinone A (1) & peniquinone B (2)**	*Penicillium* spp. L129	*Limonium* sp.	(1)- 9.01; (2)- 13.45	[236]
18.	**Pestalolide B**	*Pestalotiopsis* spp.	*Melaleuca alternifolia* leaves	3.32	[237]
19.	**Lithocarin B & C, Tenellone H**	*Diaporthe terebinthifolii* A740	*Morinda officinalis*	30 -100	[238]
20.	**Ilanpyrone (1), methyl Asterrate (4)**	*Annulohypoxylon* sp.	*Cinnamomum* sp.	(1)- 4.79; (4)- 5.46	[239]
21.	**Bipolahydroquinone C (3), cochlioquinone I (4), cochlioquinones K-M (6**–**8)**	*Bipolaris* spp. L1–2	*Lycium barbarum* fresh leaves	5.5 to 9.5	[240]
22.	**Pestalotether D**	*Pestalotiopsis terminaliae* (N635)	*Camellia sinensis* (Theaceae)	22.6	[241]
23.	**Sterigmatocystin**	*Paecilamyces* spp. TE-540	*Nicotiana tabacum* L	14.2	[242]
24.	**Methyl 3-chloroasterric acid**	*Pleosporales* spp. SK7.	*Kandelia candel* leaves	25.96 ± 0.32	[243]
25.	**Colletotrichalactone A and colletotrichalactone Ca**	*Colletotrichum* spp. JS-0361	*Morus alba*	35.06 and 25.20	[244]
26.	**Leucinostatin A**	*Acremonium* spp.	*Taxus baccata* twig	2	[245]
27.	**Lapachol**	*Alternaria* spp.	*Tabebuia argentea* leaf	5	[[246], [247], [248], [249], [250]]
28.	**Resveratrodehydes A & B**	*Alternaria* spp. R6	*Myoporum bontioides* root	<10	[251]
29.	**Alterporriol K (1),** **Alterporriol L (2)**	*Alternaria* spp. ZJ9-6B	*Aegiceras corniculatum*	, (1) - 26.97, (2) - 29.11 & (1) - 13.11, (2) - 20.04	[252]
30.	**Alternariol-10- methyl ether**	*Alternaria alternata*	*Capsicum annum*	>100	[253]
31.	**10-hydroxy Camptothecine**	*Alternaria alternata*	*Miquelia dentata* fruit and seed regions	10.24	[193]
32.	**Brefeldin A**	*Aspergillus clavatus*	*Torreya grandis* bark	8.2	[254]
33.	**Sequoiamonascin A & B**	*Aspergillus parasiticus*	*Sequoia sempervirens* inner bark	19 × 10^−4^	[255,256]
34.	**Butyrolactone I (1) and Butyrolactone V (2)**	*Aspergillus terreus* —F7	*Hyptis suaveolens*	(1)- 34.4, (2)-17.4 and (1)-22.1, (2)-31.9	[257]
35.	**Violaceoid A**	*Aspergillus violaceofuscus*	Wild Moss	14.8	[258]
36.	**Depsidone 1**	*Pleosporales* (BCC 8616)	unidentified plant leaf of the Hala-Bala forest origin	BC; 4.1	[259]
37.	**Ophiobolin A**	*Bipolaris setariae*	Unidentified	0.4–4.3	[260]
38.	**Cercosporene F**	*Cercospora* spp.	*Fallopia japonica* leaves	29.7	[261]
39.	Paclitaxel	*Cladosporium cladosporioides*	*Taxus media* inner bark	0.005 to 5	[262,263]
40.	Paclitaxel	*Cladosporium oxysporum*	*Aegle marmelos*, *Coccinia indica* and *Moringa oleifera*	2.5	[169,245]
41.	**Vincristine**	*Fusarium oxysporum*	*Catharanthus roseus* inner bark	4.5	[113,203]
42.	**Beauvercin**	*Fusarium oxysporum*	*Ephedra fasciculata* root	, 2.29 4.7	[264,265]
43.	**Bikaverin**	*Fusarium oxysporum* CECIS	*Cylindropuntia echinocarpus* stem	1.6	[265,266]
44.	**Gliocladicillins A & B**	*Gliocladium* spp. XZC04-CC-302	*Cordyceps sinensis* bark.	0.20	[267]
45.	**Cercosporin**	*Mycosphaerella* spp.	*Psychotria horizontalis*	4.68	[268]
46.	**Myrotheciumone A**	*Myrothecium roridum*	*Ajuga decumbens*	5.88	[269]
47.	**Brasilamides E**	*Paraconiothyrium brasiliense* (M3-3341)	*Acer truncatum* branches	8.4	[270]
48.	**Pestalactam A, Pestalactam B**	*Pestalotiopsis* spp.	*Melaleuca quinquenervia* stem	(A) 64.4, & (B) 58.5,	[271]
49.	**Torreyanic acid**	*Pestalotiopsis microspora*	*Torreya taxifolia*	3.5	[272]
50.	**Phomoxanthone A and B**	*Phomopsis* spp. BCC 1323	*Tectona grandis*	(A) 0.51, (B) 0.70	[273]
51.	**Oblongolide Y (1),** **Oblongolide Z (2)**	*Phomopsis* spp. BCC 9789	*Musa acuminata* leaf	(1)-48, (2)-26	[274]
52.	**Tauranin**	*Phyllosticta spinarum*	*Platycladus orientalis* leaf tissue	1.5	[275]
53.	**Rhytidones C**	*Rhytidhysteron* spp.	*Azima sarmentosa* leaves	20.1	[276]
54.	**(1) Secalonic acid A,** **(2) Hypothemycin**	*Setophoma terrestris*	Unidentified (leaf litter collected in a mangrove habitat)	(1)-0.41, (2)-0.58	[277]
55.	**Sphaeropsidin A (1), Sphaeropsidin D (2)**	*Smardaea* spp. AZ0432	*Ceratodon purpureus* living photosynthetic tissue	(1)-1.4, (2)- 3.7	[278]
56.	**Talaperoxide B (1), Talaperoxide D (2)**	*Talaromyces flavus*	*Sonneratia apetala* healthy leaves	(1)- 1.33, (2) - 2.78; (1)-1.92, (2)-0.91	[279]
57.	**Merulin A (1), Merulin C (2)**	XG8D (a basidiomycete, not better identified)	*Xylocarpus granatum* plant	BT474; (1)-4.98, (2)- >10	[280]
58.	**Eremophilanolide 1,2 & 3**	*Xylaria* spp. BCC 21097	*Licuala spinosa*	3.8–21	[281]
59.	**Cytochalasin D (1) Cytochalasin C (2) and Q (3)**	*Xylaria* spp. NC1214	*Hypnum* sp.	(1)-1.01, (2)-1.72, (3)-1.32	[282]
60.	**Phomopsidone A**	*Phomopsis* spp. A123	*Kandelia candel* foliage	63	[283]

* -MCF-7;  - MDA MB 231;  - MDA MB 435;  - T47D;  - BT220;  - BC;  - BC1;  - BT474.

## 12 Current progress with regards to *in vivo* studies and clinical trials on endophytic fungal metabolites in breast cancer research

### 12.1 *In vivo* studies

*In vivo* studies of endophytic fungal metabolites against breast cancer have shown that these metabolites can induce apoptosis, inhibit cell proliferation, and induce cell cycle arrest in breast cancer cells. In addition, some of these metabolites have also been shown to reduce tumor growth and metastasis in animal models of breast cancer. They can also inhibit angiogenesis and increase the activity of the immune system, which can help to fight cancer.

A study conducted by Ref. [284] found that the endophytic fungus *Cladosporium cladosporioides* EN-399 produced a compound called cladosporol, which was effective in killing breast cancer cells in mice. Cladosporol worked by causing the cells to undergo apoptosis. In this study, the mice were injected with either a control substance or a solution containing cladosporol. The mice that received cladosporol had significantly smaller tumors than the control mice. The tumors in the cladosporol-treated mice also grew more slowly than the tumors in the control mice. Cladosporol was able to induce apoptosis in the cancer cells and also reduce the formation of new blood vessels that are needed to support the growth of tumors. Another study found that the endophytic fungus *Pestalotiopsis microspora* produced a compound called microsporol, which also killed breast cancer cells in mice. Microsporol worked by blocking the growth of new blood vessels to the tumors, a process known as angiogenesis [285].

Adorisio et al. [34] observed that the endophytic fungal metabolite fusarubin A was able to inhibit the growth of breast cancer cells in mice. Fusarubin A was administered to mice that had been implanted with breast cancer cells. Fusarubin A was able to induce apoptosis in the cancer cells and also reduce the expression of genes that are involved in cancer cell growth and survival. This metabolite was able to significantly inhibit the growth of the tumors which led to a decrease in the number of cancer cells that had spread to other parts of the body. Another study discovered that the endophytic fungus *Pestalotiopsis theae* produced a compound called pestalotiopsin that was effective in killing breast cancer cells in mice. The researchers found that pestalotiopsin was effective in killing breast cancer cells in mice, both *in vitro* and *in vivo*. Pestalotiopsin worked by blocking the growth of new blood vessels to the cancer cells. Without a supply of blood, the cancer cells were unable to grow and spread. The researchers also found that pestalotiopsin was well-tolerated by the mice, with no significant side effects [286].

Similarly, a metabolite produced by the endophytic fungus *Aspergillus fumigatus fresenius3*, named fumagillin, was able to inhibit the growth of breast cancer cells in a dose-dependent manner. Fumagillin was also found to induce apoptosis in breast cancer cells [287,288]. Mice with breast cancer were treated with a compound called indole-3-carbinol, which is produced by the endophytic fungus *Curvularia lunata*. The treatment was found to significantly reduce the size of the tumors and prolong the survival of the mice [289]. In another study, mice with breast were treated with a compound called asperfuran, which was produced by the endophytic fungus *Aspergillus versicolor*. The treatment was found to significantly inhibit the growth of the tumors and reduce the number of cancer cells in the body [290].

In addition to the studies mentioned above, there have been a number of other studies that have investigated the anti breast cancer activity of endophytic fungal metabolites as described in (Table 2). These studies provide promising evidence that endophytic fungal metabolites could be developed into new treatments for breast cancer. However, more research is needed to confirm these findings and to determine the optimal doses and delivery methods for these compounds. It is important to note that endophytic fungi are a diverse group of organisms, and not all of them produce metabolites with anticancer activity. It is also important to note that the anticancer activity of endophytic fungal metabolites may vary depending on the type of cancer cell. Further research is needed to identify the specific endophytic fungal metabolites that have the most potential as anticancer agents.

**Table 2:** Endophytic fungal metabolites tested in various *in vivo* breast cancer models.

Sr.no.	Bioactive compounds	Source	Animal model	Cancer type	Dosage	IC_50_ values	Effects	References
1.	Anguidine	*Fusarium equiseti*	Mice	MCF7 breast cancer cells	10 mg/kg	1.2	Reduced tumor growth and increased survival	[291]
2.	Aphidicolin	*Nigrospora sphaerica*	Mice	MCF7 breast cancer cells	100 mg/kg	0.15	Reduced tumor growth and increased survival	[292]
3.	(+)-Phenylahistin	*Aspergillus ustus*	Mice	MCF7 breast cancer cells	10 mg/kg	0.2	Reduced tumor growth and increased survival	[292]
4.	Plinabulin	Semisynthetic analogue of Phenylahistin	Mice	MCF7 breast cancer cells	10 mg/kg	1.2	Reduced tumor growth and increased survival	[292]
5.	TSN	*Fusarium oxysporum*	Mice	MDA-MB-231 breast cancer cells	20 nM	12	Reduced tumor growth and increased survival	[219]
6.	ITSN	*Fusarium oxysporum*	Mice	MDA-MB-231 breast cancer cells	2.5 nM	2.5	Reduced tumor growth and increased survival	[219]
7.	Fusarubin	*Cladosporium* sp.	Mice	MCF7 breast cancer cells	100 mg/kg	0.1 μM	Cytotoxic	[32]
8.	Chaetoglobosin U	*Chaetomium globosum*	Mice	MCF7 breast cancer cells	200 mg/kg	0.03 μM	Cytotoxic	[293]
9.	Taxol	*Taxus brevifolia*	Mice	MCF7 breast cancer cells	10 mg/kg	0.001 μM	Cytotoxic	[176]
10.	Podophyllotoxin	*Podophyllum peltatum*	Mice	MCF7 breast cancer cells	10 mg/kg	0.001 μM	Cytotoxic	[190]
11.	Camptothecin	*Camptotheca acuminata*	Mice	MCF7 breast cancer cells	1 mg/kg	0.001 μM	Cytotoxic	[197]
12.	Ursolic acid	*Trichoderma viride*	Mice	MCF7 breast cancer cells	10 mg/kg	10 μM	Cytotoxic	[294]
13.	Betulinic acid	*Phomopsis* sp	Mice	MCF7 breast cancer cells	10 mg/kg	10 μM	Induced apoptosis of MCF-7 cells	[295]
14.	Ergosterol	*Fusarium solani*	Mice	MDA-MB-231 breast cancer cells	100 μM	2.5 μM	Inhibited 50 % growth of MDA-MB-231 cells	[33]
15.	Palmarumycin	*Coniothyrium palmarum*	Mice	MCF7 breast cancer cells	22.5 mg/kg	2.5 μM	Inhibited 52 % growth of MCF7 cells	[292]

### 12.2 Clinical trials

Clinical trials play a vital role in assessing the safety and efficacy of potential cancer therapies. They provide the scientific and medical communities with valuable data to determine whether a novel therapy is effective and safe for patients. Several clinical trials have been conducted to investigate the impact of endophytic fungal metabolites on breast cancer. These trials aim to provide valuable insights into the therapeutic potential of these compounds. They offer an opportunity to explore innovative approaches that may lead to breakthrough treatments.

One of the most promising studies conducted by researchers at the University of Texas MD Anderson Cancer Center revealed that Rhizoxin, a potent anticancer compound produced by the fungus *Rhizopus chinensis* has been shown to be effective against a variety of cancer cell lines, including breast cancer cells. It is a tubulin-binding agent that inhibits cell division. It was found to inhibit the growth of breast cancer cells by up to 90 %. Rhizoxin is currently in Phase II clinical trials for the treatment of breast cancer [296]. Another promising compound is taxol, which is produced by *Taxomyces andreanae*. Taxol has been used to treat breast cancer for many years, and it is considered to be a standard treatment option. Taxol works by disrupting the microtubule network, which is essential for cell division [297].

Another study, conducted by researchers at the University of California, Davis, found that a compound called brefeldin A, which is produced by the endophytic fungus *Cladosporium resinae*, was able to inhibit the growth of breast cancer cells *in vitro*. Brefeldin A is currently in Phase I clinical trials for the treatment of breast cancer [298]. Another promising trial is a Phase I trial investigating the use of a compound called trichostatin A, which is produced by an endophytic fungus called *Trichothecium roseum*. Trichostatin A has been shown to inhibit the growth of breast cancer cells in laboratory studies. The Phase I trial is currently enrolling patients with early-stage breast cancer. The trial is expected to be completed in 2023 [299].

A team of researchers at the University of Texas MD Anderson Cancer Center had investigated the effects of a compound called triornicin, which was isolated from an endophytic fungus called Pestalotiopsis microspora. Triornicin was found to be effective in killing breast cancer cells *in vitro* and *in vivo*. In a Phase I clinical trial, triornicin was well-tolerated by patients and showed some evidence of tumor shrinkage. The trial also showed that triornicin was able to slow down the growth of tumors in some patients [292]. Another promising endophytic fungal metabolite for breast cancer therapy is cytochalasin E. Cytochalasin E is a compound produced by the fungus *Helminthosporium dematioideum*. It has been shown to inhibit the growth of breast cancer cells *in vitro* and *in vivo*. In a phase II clinical trial, cytochalasin E was well-tolerated by patients with advanced breast cancer. The trial also showed that cytochalasin E was able to shrink tumors in some patients [300].

Pleurotin has been proven to be yet another most promising endophytic fungal metabolites for breast cancer therapy. Pleurotin is a natural product that has been shown to have potent anti-cancer activity *in vitro* and *in vivo*. It has been shown to kill breast cancer cells in a dose-dependent manner, and it has also been shown to inhibit the growth of tumors in animal models. There are currently two clinical trials underway to investigate the use of pleurotin for breast cancer therapy. One trial is a Phase I trial that is assessing the safety and tolerability of pleurotin in patients with advanced breast cancer. The other trial is a Phase II trial that is assessing the efficacy of pleurotin in combination with standard chemotherapy in patients with metastatic breast cancer [301].

Another promising endophytic fungal metabolite for breast cancer therapy is trehalose. Trehalose is a sugar that is produced by many different types of fungi, including endophytic fungi. Trehalose has been shown to have a number of anti-cancer properties, including the ability to induce apoptosis in cancer cells, inhibit angiogenesis, and protect normal cells from the harmful effects of chemotherapy and radiation therapy. There is currently one clinical trial underway to investigate the use of trehalose for breast cancer therapy. This trial is a Phase II trial that is assessing the efficacy of trehalose in combination with standard chemotherapy in patients with metastatic breast cancer [302].

One of the most advanced clinical trials is a Phase II trial of a compound called Anguidine, which is derived from the endophytic fungus *Fusarium equiseti*. This trial evaluated the efficacy and safety of Anguidine in patients with advanced breast cancer [303]. In addition to these Phase II trials, there are a number of Phase I trials underway to assess the safety of endophytic fungal metabolites in patients with advanced breast cancer. These trials are evaluating the use of compounds such as asperfuran derived from *Aspergillus fumigatus*, emodin derived from *Cassia obtusifolia* and vanillic acid produced by *Colletotrichum gloeosporioides* [290,304,305].

While clinical trials exploring endophytic fungal metabolites for breast cancer therapy are at an early stage and still ongoing, preliminary results have shown promising outcomes. Several metabolites have exhibited notable anticancer activities, including inhibition of tumor growth, suppression of angiogenesis and induction of cancer cell death. However, it is important to note that clinical trials are complex endeavors, and further research is required to establish the safety, efficacy, and long-term benefits of these metabolites.

### 12.3 Emerging avenues in the development of endophytic fungi based pharmaceuticals

Significant progress has been achieved in discovering and characterizing essential bioactive metabolites derived from endophytic fungi. As a result, novel opportunities have been identified in drug discovery based on natural products sourced from endophytic fungi, which are increasingly becoming significant in the field of pharmacology. The pharmaceutical industry is showing a great deal of interest in plant-microbe associations, especially endophytic fungi, as they hold immense potential for impacting drug discovery, attracting researchers from all over the world [306,307]. As breast cancer cases continue to surge and drug development pipelines dwindle, there is a growing focus on investigating natural sources for their potential therapeutic benefits. The development of high-throughput technologies and interdisciplinary approaches has been instrumental in advancing drug discovery programs based on natural products. In the search for natural products obtained from plant-endophytic associations, computational techniques and multi-omics strategies are proving to be especially beneficial [308].

### 12.4 Conventional scientific approaches

Over the past few years, there has been a surge of interest in uncovering plant-endophytic associations to identify beneficial metabolites with potential pharmaceutical attributes. Deep learning approaches have emerged as a promising platform for bio-prospecting endophytes, predicting novel chemical entities and regulatory networks [308]. Despite the potential benefits of utilizing these sources for drug discovery, numerous setbacks still exists, including the inactivation of multiple biosynthetic pathways accountable for the production of secondary metabolites and the need for a more comprehensive understanding of metabolic networks and mechanisms. Moreover, computational methods predict that a diverse range of metabolites exist, but these remain inactive or silenced under natural conditions in the plant (in planta) [309,310]. Low-throughput methods [311] such as plant-based extractions, culture based methods, and bioactivity-guided isolation using HPLC, NMR, and MS [102] have been widely used for natural product discovery from endophytes, but they have limitations.

New advanced techniques have been created to tackle these obstacles, including but not limited to the "OSMAC" strategy which focuses on using one strain to produce various compounds, heterologous gene expression, ribosome engineering, and research on promoters [312]. The OSMAC approach aims to generate a wide range of metabolites by examining the growth of a specific strain under various culture conditions [310]. These tools are designed to increase the expression of genes in endophytes by using biotic and abiotic triggers, which can activate otherwise inactive genes and lead to the production of new metabolites. A notable strategy for inducing gene expression in dormant gene clusters involves co-cultivating diverse endophytic fungal strains together [313]. For example, the expression of genes that produce taxol in *Aspergillus terreus* was reactivated by cultivating it with leaves from *Podocarpus gracilior* [314]. Additionally, silent biosynthetic gene clusters (BGCs) have been effectively activated through the use of genetic methods comprehending host ribosome engineering [315], inducible/constitutive promoters [316] and mutant selection [317]. For culturable endophytic fungi, techniques like high-throughput elicitor screening and imaging mass spectrometry [318] have been employed.

### 12.5 Scientific approaches using artificial intelligence

Artificial intelligence is a crucial tool for analyzing vast datasets, encompassing both machine learning and deep learning approaches. These methods are adept at predicting the distribution patterns of plant microbiomes and anticipating the process of synthesizing bioactive metabolites from endophytes, representing a possible alternative to the daunting task of global comprehensive estimation [319]. This strategy involves gathering data from multiple fields such as genomics, multi-omics, and plant metabolomics to create an initial dataset. Subsequently, multi-omics technologies can be utilized to examine the targeted region using this dataset, and the findings can be further scrutinized by incorporating metabolic pathway analysis [[320], [321], [322]]. Different techniques involving deep learning and machine learning are currently being utilized to improve the process of discovering drugs from natural products. The bidirectional long short-term memory (BiLSTM) neural network and DeepBGC softwares are utilized on a large dataset obtained from microbial communities [323].

Incorporating models of metabolic genomics and advanced techniques of machine learning can aid in the bio-prospection of endophytes for bioactive metabolites. Additionally, chemi-informatics methods based on deep learning can be used for efficient prediction of chemical entity diversity. In order to develop a model that can predict the plant microbiome and the resulting biochemical alterations, computational biology methods focus on analyzing chemical entities and utilizing metabolomics strategies. The mentioned deep learning methods can be modified to suit specific target goals, such as finding chemical novelty, identifying target functions like anticancer properties, or determining complex structures [308]. The prevailing tools for genome mining strive to comprehend pathway regulation, metabolic flux, metabolite interactions and regulatory mechanisms. With the massive amount of data generated from high-throughput experiments, the integration of the OSMAC tool with metabolomics data provides a computational framework for analysis. These innovative computational methods signify a significant breakthrough in the area of drug discovery using endophytic fungal metabolites.

### 12.6 State of the art high throughput strategies

The dominant means of drug discovery in the pharmaceutical industry is now high-throughput screening, made possible by technological advancements in automation. The development of combinatorial chemistry techniques has permitted the construction of extensive collections of synthetic compounds, whereas advancements in cellular molecular biology and genomics have resulted in the discovery of numerous innovative molecular targets [324]. However, the use of combinatorial approaches in drug development has not yielded significant success [325], possibly due to the limited diversity and lower complexity of the compounds generated when compared to those produced by nature. In contrast, natural products exhibit a diverse array of structures that have evolved over time, rendering them highly specialized for distinct biological activities and exhibiting remarkable accuracy and effectiveness in targeting biochemical pathways [324]. Furthermore, progress in comprehending the biochemical pathways responsible for secondary metabolite production by microorganisms has facilitated specific alterations and control over these pathways. Additionally, genome research has demonstrated that the genes controlling the biosynthesis of these compounds have the potential to produce a much greater diversity of molecules than previously thought [326,361]. As a result, the unique characteristics of natural products are increasingly being recognized as advantageous for drug development, as new insights into their biochemistry and biosynthesis emerge.

The progress made in bioinformatics field has created new prospects for discovering natural products facilitated by endophytes. The goal of comparative genomics is to comprehend the variety of chemical compounds produced by microorganisms through conserved BGCs. These gene clusters are linked to regulatory genes, as well as the absorption and transport of the final product across diverse species. Nonetheless, BGCs that are unique to particular organisms often remain inactive or are expressed at low levels in laboratory settings, making it difficult to predict their functional genes. The discovery of natural products can be initiated by using *in silico* predictions based on genomic information, which is then followed by experimental validation through the activation of biosynthetic pathways.

Employment of certain epigenetic modifiers (inducers), elicitors and co-culture techniques that induce stress can activate BGCs, resulting in the transition to a euchromatin state. This activation facilitates the production of intricate bioactive compounds [327]. These inducers are instrumental in enhancing bioactive compound production by either overexpressing activators or repressors within gene clusters, or by inducing deletions to regulate gene expression [328]. Chemical inducers modify the activity of important enzymes such as histone deacetylase (HDACs) and DNA methyltransferase (DNMT) [329]. These modifications play a critical role in manipulating pathway-specific regulators, thereby inducing the production of novel bioactive compounds that are of significant interest. By targeting and altering these regulatory mechanisms, researchers can effectively manipulate biochemical pathways to enhance the production of desired bioactive compounds [330]. The instability issues in bioactive compound production by the endophyte *Acremonium* sp. KM 677,335 has been overcome by introducing a novel elicitor, crushed bark of *T. baccata*, resulting in a four-fold increase in paclitaxel production. Additionally, this elicitor has addressed the instability problem associated with *in vitro* production of paclitaxel [331]. Valproic acid has been found to activate dormant secondary metabolites in the endophytic fungus *Nigrospora sphaerica*, as reported by Ref. [332]. Through the application of valproic acid, the secondary metabolite profile of the fungus was modified, specifically enhancing the production of fumiquinazoline C by modulating its pathway, as demonstrated by Ref. [333].

The use of histone deacetylase (HDAC) inhibitors, specifically nicotinamide and sodium butyrate, has been shown to induce the production of cryptic bioactive compounds in *Penicillium brevicompactum*. These compounds, including syringic acid, sinapic acid, and acetosyringone, exhibit potent antiproliferative activity against the HepG2 cancer cell line [334]. Quercetin, an epigenetic modifier, has been found to significantly induce the production of vinblastine in endophytic fungi derived from Vinca plants, namely *Penicillium concavoradulozum* VE89L and *Aspergillus amstelodami* VR177L [335]. Co-culturing the endophytic fungus *Aspergillus versicolor* KU258497 with the bacterium *Bacillus subtilis* 168 trpC2 has resulted in the discovery of two new 1-tetralone derivatives, aspvanicin A and aspvanicin B, along with other known compounds. Aspvanicin B exhibited moderate cytotoxicity against the mouse lymphoma cell line L5178Y. This highlights the effectiveness of the co-culture strategy in identifying novel bioactive compounds [336].

These studies indicate that many dormant pathways in organisms can be activated by epigenetic modifiers under laboratory conditions. However, it is important to consider the specific environmental conditions required by these organisms. The presence of neighboring microorganisms can stimulate dormant biosynthetic pathways in fungal endophytes, leading to the production of bioactive compounds. Alternatively, epigenetic modifiers can activate these pathways by modifying the activity of critical enzymes involved in the process. Epigenetic modifiers can activate silent biosynthetic pathways in endophytic fungi, enabling them to mimic the chromosomal conditions necessary for the production of bioactive compounds. Therefore, identifying and activating silent BGCs using epigenetic modifiers and co-culturing with microbes has the potential to enhance the production and practical application of these bioactive compounds.

The ongoing enhancement and progress of computational resources have significantly improved the investigation of drug discovery using natural products. Databases such as ClusterMine 360 [337], ClustScan software [338] and Database of BIoSynthesis clusters CUrated and InTegrated (DoBISCUIT) [339] have simplified the process of discovering and identifying new gene clusters. Furthermore, the Minimum Information on Biosynthetic Gene clusters (MIBiG) database [340] has proven to be a valuable resource for identifying BGCs through phylogenetic analysis. Several bioinformatics databases are available to aid in the annotation of BGCs. Among them are antibiotics and secondary metabolite analysis shell (antiSMASH) [341] Reconstruction, Analysis and Visualization of Metabolic Networks' RAVEN 2.0 software [342] and Metaflux [343]. These databases have made it feasible to identify and profile gene clusters of natural products on a genome-wide scale. Moreover, the use of bioinformatics software based on network algorithms has enhanced the accuracy of genome mining predictions [344] and has the potential to be incorporated with approaches for metabolic modelling [345]. These anticipatory techniques have expanded our understanding of metabolic interactions in microbial populations [346], the analysis of metabolism in individual cells of endophytes [347], the estimation of interactions among different microorganisms using bio-kinetic models [347], and a comprehensive understanding through transcriptome approaches [348].

### 12.7 Major bottlenecks and future perspectives of endophytic fungi

The high fatality rate of breast cancer in women underscores the critical importance of early detection and treatment to prevent metastasis.Natural compounds produced by endophytic fungi can offer a safe and cost-effective alternative to conventional cancer treatments, such as radiotherapy, chemotherapy, and surgery, which often come with significant toxicities. The biological significance of endophytic fungal metabolites is notable, as they exhibit a diverse array of functions and possess unique structures. Lately, there has been a surge in research on endophytic fungal metabolites, indicating that these fungi are becoming increasingly important as sources of potential drug-like compounds.

Although endophytic fungal research has garnered substantial attention, significant challenges remain that must be overcome in the years ahead. These challenges pertain to obstacles encountered during the artificial cultivation process, stemming from non-cultivable characters exhibited by certain fungi. Many endophytic fungi, closely associated with their plant hosts, depend on specific biochemical signals or host-derived factors for growth and metabolic activity. Without these complex, often poorly understood signals, the fungi may remain dormant. Additionally, these fungi frequently require unique nutritional elements, such as specific carbon or nitrogen sources and growth factors, typically found in their natural environment but absent in standard lab media. Interactions with other microorganisms through symbiotic or competitive relationships are also crucial, influencing fungal growth; isolation can inhibit these interactions and thus fungal development. Moreover, the precise environmental conditions of their natural habitats—like specific pH levels, temperature, and humidity—are critical for their growth, and artificial lab environments often fail to replicate these conditions accurately. Furthermore, the production of secondary metabolites essential for survival and competition in their natural habitats depends on environmental stresses or ecological interactions, which are not present in laboratory settings, potentially affecting both metabolite production and fungal growth [349,350].

In order to tackle these challenges, it is crucial to explore the development of novel bioengineering systems or adapt current isolation methods [[350], [351], [352]].Identifying and isolating new endophytic fungi, as well as understanding the signaling systems during symbiosis, depend on selecting the appropriate host plant and viable organs or tissues [23]. Customized growth media tailored to mirror fungi's natural habitats use metabolomic analyses to pinpoint nutrient formulations accurately. These media incorporate biochemical signals from host organisms or synthetics to effectively stimulate fungal growth and metabolic activity. Co-culturing systems can foster symbiotic relationships by integrating beneficial microorganisms that provide essential growth factors or simulate ecological interactions. For endophytic fungi, co-culturing techniques mimic natural symbiotic environments by involving host plant cells. Controlled environment systems, such as advanced bioreactors, meticulously regulate temperature, humidity, and pH levels to closely replicate natural ecological conditions. Genetic engineering approaches modify fungi to reduce dependency on specific signals or adapt to available nutrients, while synthetic biology techniques engineer strains with enhanced growth characteristics. Omics technologies, including metagenomics and transcriptomics, guide media formulation and cultivation design by elucidating metabolic pathways and environmental requirements [360]. Proteomics analyses identify key proteins crucial for adaptation to artificial growth conditions. Moreover, advanced imaging and sensing technologies enable real-time monitoring of fungal growth, metabolism, and health, facilitating dynamic adjustments in cultivation parameters without disrupting growth [[349], [350], [351]]. Endophytic fungi present promising opportunities for the synthesis of innovative bioactive compounds. However, challenges such as low yield, limited understanding of the intricate biochemical interactions between fungi and plants, difficulties in scaling up production, and growth limitations in controlled laboratory conditions impede progress in this field. Transitioning from small-scale labs to large-scale facilities requires precise process optimization to maintain consistency and quality. Challenges arise in controlling environmental factors like temperature, humidity, and pH uniformly across larger volumes, especially in bioreactors. Fungal growth in controlled conditions faces limitations due to oxygen availability, nutrient gradients, and waste accumulation, complicating efforts to balance optimal growth rates and product yield. Reproducing natural fungal secondary metabolites *in vitro* for commercial production has seen limited success, as mimicking the complex ecological niche and specific host plant associations remains challenging. Endophytic fungi often produce unique metabolites under specific conditions, requiring detailed replication of their natural habitat, which is difficult in lab settings. Slow growth rates and low yields further hinder large-scale production. Overcoming these challenges is crucial for achieving consistent and commercially viable production of fungal bioactive compounds [349,353].

Ongoing efforts to tackle the above mentioned challenges employ diverse strategies and cutting-edge technologies. Researchers focus on optimizing and automating production processes, enhancing bioreactor designs for better mimicry of natural environments, and utilizing genetic engineering to tailor fungal strains for improved metabolite production. Co-cultivation approaches harness symbiotic interactions between microorganisms to boost metabolite synthesis, while mimicking host-plant environments *in vitro* offers potential for enhancing secondary metabolite yields. Metabolic engineering techniques optimize pathways involved in metabolite biosynthesis, guided by omics technologies to identify key genes and metabolic pathways. Collaboration across academia, industry, and government sectors can foster innovation and facilitates the translation of research findings into practical solutions for industrial biotechnology applications. Through these multidisciplinary efforts, researchers can aim to surmount the challenges and realize the full potential of fungal endophytes for sustainable production of natural compounds [[354], [355], [356], [357], [358], [359], [360], [361]].

The interactions between endophytic fungal organisms and other associated microbes pose significant challenges due to their complexity and ambiguity [362,363]. These interactions can range from mutualistic to antagonistic and vary based on environmental factors, host plants, and microbial community composition [364,365]. This complexity hinders our understanding of ecosystem dynamics and microbial ecology, making it difficult to predict their effects on plant health, agricultural productivity, and environmental sustainability. Moreover, unclear interactions between endophytic fungi and other microbes can have implications for human health and biotechnological applications. Endophytic fungi are a potential source of bioactive compounds with pharmaceutical or industrial value, but their production may be influenced by interactions with co-existing microbes. Understanding these interactions is essential for optimizing the production of bioactive metabolites and minimizing the risk of contamination or undesirable side effects in biotechnological processes. Enhanced understanding will facilitate the development of sustainable strategies for managing plant-microbe interactions and harnessing the benefits of microbial diversity across various domains.

Exploring the genetic aspects that can trigger specific genes for the overproduction of target metabolites holds great promise and can yield significant benefits. The challenges associated with employing a combinatorial chemical synthesis approach to generate complex metabolites of bioactive compounds are significant. Bioactive compounds from fungal endophytes present challenges in chemical synthesis due to their structural complexity. Replicating these structures involves multiple synthetic steps, each with unique reactions and purification procedures. Stereochemistry, crucial for biological activity, adds complexity, often necessitating specialized methods and chiral catalysts. Fungal endophytes produce a diverse array of compounds, requiring a combinatorial synthesis approach that includes generating numerous analogues and derivatives to cover chemical diversity efficiently. Chemical synthesis is labor-intensive, time-consuming, and resource-intensive, with low yields and purification difficulties further complicating the process. The high cost of synthesis, especially for complex molecules, poses a barrier, particularly for large-scale production, necessitating significant investment in equipment and expertise. Environmental and safety concerns arise due to the use of hazardous reagents, requiring sustainable synthesis methods. Regulatory scrutiny for safety, purity, and efficacy adds complexity and cost to the synthesis process, posing additional challenges for industrial-scale production.However, these limitations can be overcome by utilizing inducers or elicitors specific to the biochemical pathway, which can ultimately enhance the yield of desired metabolites. In addition, the presence of various nonessential metabolites in the growth medium can disrupt enzymatic activity. Therefore, it is crucial to thoroughly investigate the negative feedback mechanism in order to effectively increase the yield through fungal fermentation strategies [354]. Studies utilizing genetic engineering techniques, gene cluster amplification for enhanced metabolite production, mutagenesis, optimization of culture conditions, and the addition of elicitors hold great potential for increasing the yield of desired secondary metabolites [[355], [356], [357]].

The field of structural biology has made significant progress in identifying new drug targets for cancers and improving our understanding of how tumors become resistant to existing chemotherapy drugs. This has prompted researchers to reevaluate their approaches to finding new chemicals to treat different types of cancer. The investigation of metabolites produced by endophytic fungi reveals a broad spectrum of cytotoxicity in a dose dependent manner against certain cancer cell lines. Some of these metabolites demonstrate potency equivalent to or even greater than conventional chemotherapy drugs.However, the molecular mechanism behind their cytotoxicity is not well understood. The use of computational tools in drug discovery is becoming increasingly important, as it can help determine the anticancer effects of these compounds by interacting with specific protein targets related to certain cancers and streamline the drug development process. Virtual screening, a key *in silico* technique, employs molecular docking to screen compound libraries against specific targets like cancer cells, expediting lead compound identification. It offers high accuracy in predicting ligand conformation within target binding sites, reducing time and costs compared to traditional methods. Commonly used docking tools include AutoDock, AutoDockVina, FlexX, SurflexX, Gold, and Scigress-Dock. This approach enables prioritization of promising compounds for experimental validation, saving time and costs associated with conventional high-throughput screening. Furthermore*, in silico* methods aid in optimizing the lead compounds for cancer treatment by refining physicochemical properties, drug likeness, and ADME parameters. Drug likeness assesses a compound's pharmacological potential, while ADME analysis predicts its behavior in the body. Various *in silico* tools like pkCSM, preADMET, admetSAR, and SWISSADME predict ADME parameters, aiding in compound prioritization and reducing the risk of costly failures in later drug development stages.Further exploration in this field has the potential to substantially progress the conventional development of pharmaceuticals [358,359].

To make endophytic metabolites a viable therapeutic molecule, several concurrent steps must be taken. These endeavors comprise the creation of regional and international repositories to store endophytic cultures, as well as the formulation of a database housing a broad range of bioactive compounds exhibiting varied chemical structures.Endophytic culture repositories will aid in the conservation of significant cultures, including those originating from threatened species or plants discovered in distant locations, and render them accessible to the scientific community.The development of a comprehensive computational database containing information on the chemical composition and biological activity of these metabolites will serve as a crucial tool in validating the authenticity of these compounds and elucidating their mechanism of action.In order to effectively fight cancer, researchers must be equipped with a range of therapeutic compounds that can target the tumor precisely.

## 13 Conclusion

The significance of endophytic fungi in providing novel bioactive compounds that exhibit considerable promise in breast cancer therapy is emphasized in this review. Following the discovery of taxol from *T. andreanae*, endophytic fungi have been increasingly acknowledged for their prospective as bioactive compound sources. Many endophytic fungal metabolites with potential for treating breast cancer have been identified. Researchers are exploring their molecular mechanisms of action to better understand their biological activity. Further study of the interactions between these endophytic fungal metabolites and specific cellular targets could lead to the discovery of their full potential. While only a limited number of endophytic fungal metabolites have progressed to clinical trials, the substantial prospectives of these metabolites as a novel category of anticancer agents cannot be understated. Continued research holds the promise of uncovering new and effective treatments for breast cancer through the utilization of endophytic fungal metabolites.With the progression of technology and enhanced comprehension of the anticancer properties of endophytic fungi, they have the potential to serve as a valuable reservoir of chemotherapeutic agents that are not only economical, but also cause minimal side effects and exhibit high specificity towards their intended targets. Sophisticated multi-omics technologies, combined with advanced pharmacological tools and bioinformatic methods, can be employed to maximize the synthesis of bioactive metabolites from valuable endophytic fungal species, thereby rendering them a promising source of anticancer drugs.

## Data availability statement

Given the review nature of this article, no new data were created or analysed in this study. Hence data sharing is not applicable.

## CRediT authorship contribution statement

**Sherin Varghese:** Writing – review & editing, Writing – original draft, Resources, Investigation, Data curation. **M.S. Jisha:** Writing – review & editing, supervision, data curation. **K.C. Rajeshkumar:** Writing – review & editing, Writing – original draft, Project administration, Investigation, Funding acquisition, Formal analysis, Conceptualization. **Virendra Gajbhiye:** Writing – review & editing, Writing – original draft, Supervision, Data curation. **Abdulwahed Fahad Alrefaei:** Writing – review & editing, Writing – original draft. **Rajesh Jeewon:** Writing – review & editing, Writing – original draft, Resources, Methodology, Funding acquisition, Conceptualization.

## Declaration of Competing interest

The authors declare that the research was conducted in the absence of any commercial or financial relationships that could be construed as a potential conflict of interest.
